# Vaccinia virus replication is not affected by APOBEC3 family members

**DOI:** 10.1186/1743-422X-3-86

**Published:** 2006-10-19

**Authors:** Melanie Kremer, Yasemin Suezer, Yolanda Martinez-Fernandez, Carsten Münk, Gerd Sutter, Barbara S Schnierle

**Affiliations:** 1 Paul-Ehrlich-Institut, Paul-Ehrlich-Str. 51–59, 63225 Langen, Germany

## Abstract

**Background:**

The APOBEC3G protein represents a novel innate defense mechanism against retroviral infection. It facilitates the deamination of the cytosine residues in the single stranded cDNA intermediate during early steps of retroviral infection. Most poxvirus genomes are relatively A/T-rich, which may indicate APOBEC3G-induced mutational pressure. In addition, poxviruses replicate exclusively in the cytoplasm where APOBEC3G is located. It was therefore tempting to analyze whether vaccinia virus replication is affected by APOBEC3G.

**Results:**

The replication of vaccinia virus, a prototype poxvirus, was not, however, inhibited in APOBEC3G-expressing cells, nor did other members of the APOBEC3 family alter vaccinia virus replication. HIV counteracts APOBEC3G by inducing its degradation. However, Western blot analysis showed that the levels of APOBEC3G protein were not affected by vaccinia virus infection.

**Conclusion:**

The data indicate that APOBEC3G is not a restriction factor for vaccinia virus replication nor is vaccinia virus able to degrade APOBEC3G.

## Background

During evolution, eukaryotic cells had to cope with a large amount of pathogens. The interaction of host and pathogen required defense responses in the host, which resulted in the development of the innate immune system. Due to this high selection pressure, pathogens developed strategies to escape or manipulate the host immune defense. *Poxviridae *in particular, evolved several mechanisms for immune evasion [1,2]. The best known members of this family are variola and vaccinia virus. Variola virus is the causative agent of smallpox, and although eradicated in the early 70s, it still represents a serious threat as a possible agent for bioterrorism. Vaccinia virus (VACV) is the prototype poxvirus and is frequently used as a vector for vaccine development. The large double-stranded DNA genome of poxviruses is 130 to 300 kb in size, and although most genomes are completely sequenced, the function of many genes necessary for viral infection, replication and immune evasion are not known [3].

APOBEC3G is a recently discovered defense mechanism against retroviral infection [4]. The protein becomes encapsidated into retroviral particles and is transported into the infected cell, where it facilitates deamination of cytosine residues in the single stranded cDNA intermediate during early steps of infection. APOBEC3G has been shown to be an exclusive DNA mutator [5]. The replacement of C with U in the DNA minus strand during reverse transcription leads to G to A transitions in the plus strand. APOBEC3G, therefore, triggers G to A hypermutations in the newly synthesized viral DNA. The inhibition of viral replication is due either to degradation of the cDNA by the DNA repair machinery or to the lethality of the hypermutations.

In addition to its anti-retroviral function [4,6], APOBEC3G is also able to restrict hepadnaviruses [7], and its gene family member APOBEC3A inhibits parvovirus replication [8]. APOBEC3G is located in the cytoplasm of the cell where it performs its function. VACV undergoes its complete viral life cycle in the cytoplasm, and most poxvirus genomes are relatively A/T-rich, which could be caused by APOBEC3G-induced mutational pressure [9]. We were, therefore, interested to determine whether APOBEC3G is also a restricting factor for this virus.

## Results and Discussion

**Figure 1:**
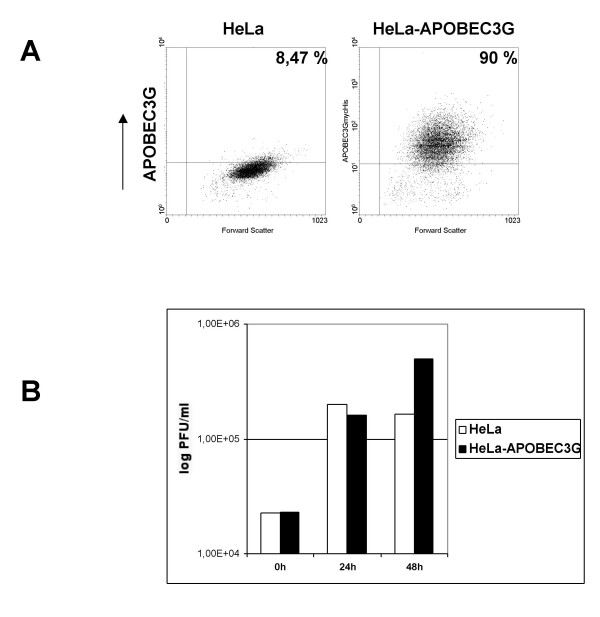
**VACV replication in APOBEC3G-expressing HeLa cells**. A: APOBEC3GmycHis is expressed in 90% of HeLa-APOBEC3G cells. Expression was confirmed by intracellular staining with a mouse anti-Myc antibody (BDBiosciences, Heidelberg) and a FITC-conjugated anti-mouse IgG antibody (Dianova, Hamburg) followed by FACS analysis. B: Viral replication is not impaired by APOBEC3G expression. HeLa-APOBEC3G and HeLa cells were infected with VACV strain WR at an MOI of 0.05 and viral titers were measured 0, 24 and 48 h post infection by titration on RK13 cells.

**Figure 2:**
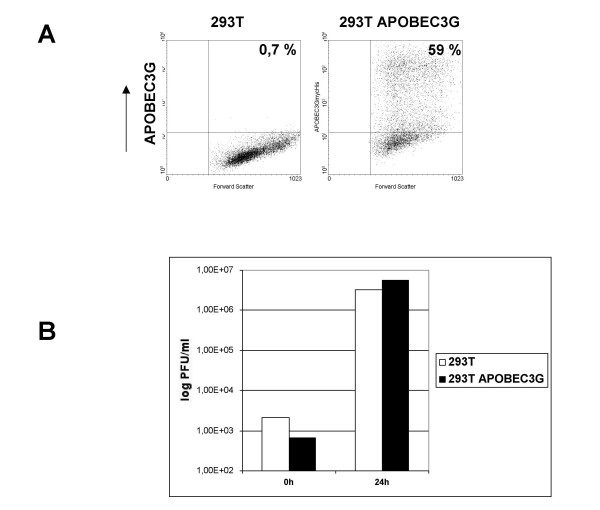
**VACV replication in 293T cells expressing APOBEC3G**. A: An APOBEC3GmycHis expression plasmid was transiently transfected into 293T cells using the Fugene reagent (Roche, Penzberg) 48 h prior to infection. Transfection efficiency was determined by intracellular staining with a mouse anti-Myc antibody (BDBiosciences, Heidelberg) and a FITC-conjugated anti-mouse IgG antibody (Dianova, Hamburg) followed by FACS analysis, which showed that 59% of cells expressed APOBEC3GmycHis after transfection. B: APOBEC3GmycHis expression does not impair viral replication in 293T cells. 293T cells and 293T cells expressing APOBEC3GmycHis were infected with VACV strain WR at an MOI of 0.05 and viral titers were measured 0 and 24 h post infection by titration on RK13 cells.

**Figure 3:**
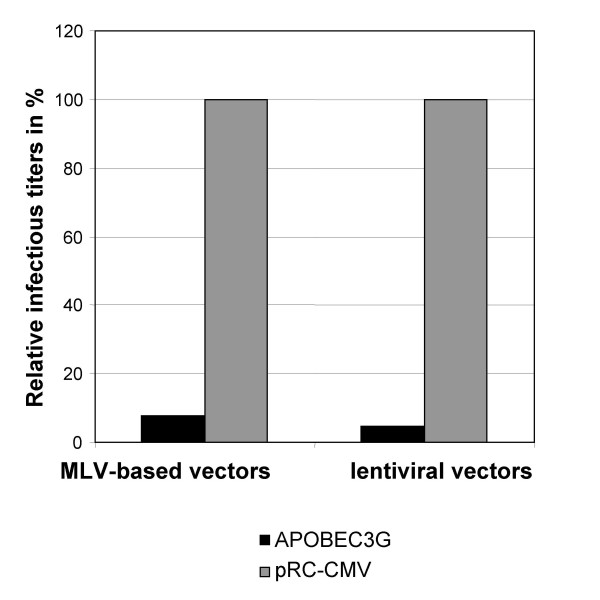
**APOBEC3G reduces retroviral and lentiviral vector titers**. Retroviral or lentiviral vectors encoding GFP were produced by transient transfections of 293T cells either in the presence of an APOBEC3G expression plasmid or the empty vector (pRC-CMV). Titers were determined by FACS analysis using NIH3T3 cells. Relative titers are given by setting the empty vector control to 100%.

To assess the impact of APOBEC3G on the VACV life cycle, we used HeLa-APOBEC3G cells which were stably transfected with an APOBEC3GmycHis expression plasmid encoding a Myc- and 6-His-tagged protein [10]. Intracellular APOBEC3G expression was confirmed by flow cytometry after staining the cells with a mouse anti-Myc antibody. About 90% of the HeLa-APOBEC3G cells expressed APOBEC3G (Figure 1A). HeLa-APOBEC3G cells were infected with VACV of the strain Western Reserve (VACV-WR) at an MOI of 0.05. This ensured continuous viral replication and viral spread throughout the cell culture. Cells were harvested and viral titers were determined 0, 24 and 48 h post infection on RK13 cells. As shown in Figure 1B, there were no differences in viral replication in HeLa-APOBEC3G cells compared to the parental HeLa cells, which do not express APOBEC3G. Titers from VACV-infected HeLa-APOBEC3G cells were even slightly higher at 48 h post infection. This indicates that APOBEC3G has no negative effect on VACV replication. To confirm the results obtained with HeLa cells, we also investigated viral replication in APOBEC3G-expressing 293T cells. 293T cells were transiently transfected with the APOBEC3GmycHis expression plasmid. The transfection efficiency was determined by intracellular APOBEC3G staining with a mouse anti-Myc antibody and flow cytometry. This demonstrated that 59% of the transfected 293T cells expressed APOBEC3GmycHis (Figure 2A). To investigate the replication of VACV, 293T cells and 293T APOBEC3G cells were infected at an MOI of 0.05 and viral titers were measured 0 and 24 h post infections by titration on RK13 cells. Again, the replication of VACV in APOBEC3G-transfected 293T cells was not altered compared to parental 293T cells (Figure 2B). As a control, to validate the experimental settings, co-transfection of APOBEC3G was used to study its inhibitory effect on retroviral and lentiviral vector transduction. Murine leukemia virus (MLV) and human immunodeficiency virus type 1 (HIV-1)-based vector particles were generated by transient transfections of 293T cells. Vector titers were determined by transduction of the green fluorescence protein (GFP)-encoding vector sequences into NIH3T3 cells and the number of GFP-positive cells was monitored by flow cytometry. Vector titers obtained by co-transfection of the empty expression vector were set to 100% (Figure 3). Co-expression of APOBEC3G drastically reduced retroviral and lentiviral vector titers (Figure 3) and validates the co-transfection system as a useful tool to study the effect of APOBEC3 proteins on viral infectivity.

**Figure 4:**
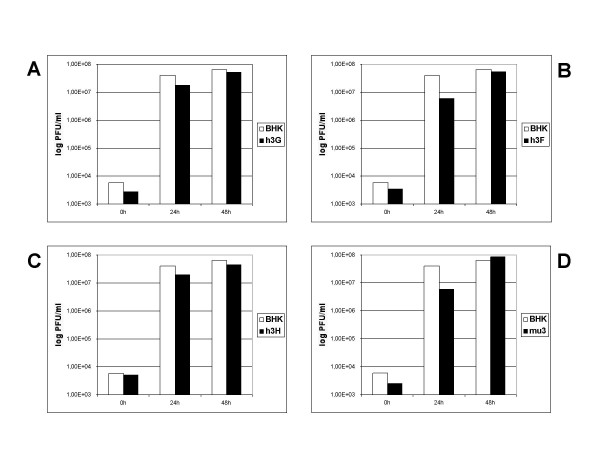
**VACV replication in APOBEC3-transfected BHK cells**. BHK cells were transfected with an APOBEC3G, -F or -H [25], or mouse APOBEC3 [26] expression plasmid 48 h prior to infection and then infected with VACV strain WR at an MOI of 0.05. Viral titers were determined 0, 24 and 48 h post infection by titration on RK13 cells and showed that replication in BHK cells was not altered by the expression of APOBEC3. A: APOBEC3G; B: APOBEC3F; C: APOBEC3H; D: mouse APOBEC3.

In addition, we sought to assess whether other members of the APOBEC3 gene family are able to constrain VACV replication. We tested the influence of APOBEC3G, -F and -H, and mouse APOBEC3 on VACV replication by transient transfection of expression plasmids into BHK cells, followed by infection with VACV-WR at an MOI of 0.05. Viral titers were measured 0, 24 and 48 h after infection by titration on RK13 cells. Although the transfection rate was usually around 50%, expression of the APOBEC3 proteins in BHK cells had no influence on VACV replication (Figure 4A–D).

**Figure 5:**
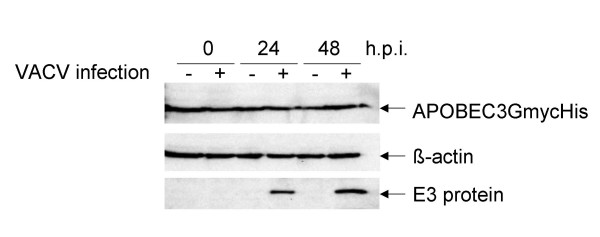
**APOBEC3G protein level is not altered by infection with VACV**. HeLa-APOBEC3G cells were infected with VACV strain WR at an MOI of 2. Cell lysates of infected and uninfected cells were obtained 0, 24 and 48 h post infection. APOBEC3GmycHis expression was analyzed by Western blot with a mouse anti-6xHis antibody (Acris, Hiddenhausen, Germany). After stripping the blot, infection was confirmed with an anti-E3L antibody (generous gift of B. Jacobs) and equal loading with an anti-β-actin antibody (Sigma-Aldrich).

During wild-type human immunodeficiency virus (HIV) infection, APOBEC3G is inactivated by the HIV accessory protein Vif, which targets it for degradation by the ubiquitin-dependent proteasomal pathway. Therefore, APOBEC3G only restricts Vif-deleted HIV [14,15,10]. Consequently, we asked whether VACV has developed a similar strategy to evade APOBEC3G. To address this issue, we infected HeLa-APOBEC3G cells with VACV at an MOI of 2 to ensure infection of all cells. Cell lysates were obtained 0, 24 and 48 h post infection and analyzed by Western blot with an antibody directed against the 6xHis-tag. VACV infection did not alter APOBEC3G protein levels, nor did it cause a degradation of the protein, which might have resulted in the appearance of smaller bands during the Western blot analysis (Figure 5). Equal loading was confirmed by detection of β-actin, and infection was proven by detection of the VACV early protein E3 after stripping the blot. The E3 protein is very stable and can still be detected at late time points of infection. These data suggest that VACV has not developed mechanisms to degrade APOBEC3G and suggests that interference with this protein is not required for VACV replication.

Poxviruses are large cytoplasmic DNA viruses and infection of a cell initiates drastic responses that aim to eliminate virus-infected cells. This innate immune response is able to restrict a multitude of viruses by various strategies. The APOBEC3 gene family contains recently discovered factors that are able to restrict retroviruses, hepadna viruses and parvoviruses [16,7,8]. The recently described inhibition of a parvovirus by human APOBEC3A shows that APOBEC proteins also target DNA viruses that replicate in the nucleus without passing through an RNA intermediate [8]. The G and F members of the human APOBEC3 family have two highly conserved zinc-binding domains, characteristic of the catalytic domain (CD) of all cytidine deaminases, and trigger G-to-A hypermutations in the newly synthesized viral DNA. The APOBEC3H cytidine deaminases domain, however, is evolutionary distinct [17]. In contrast to primates, rodents encode only one APOBEC3 protein. This protein cannot inhibit the murine leukemia virus (MLV) but, like APOBEC3G and -F, it is able to restrict HIV-1 [18-20]. Human APOBEC3H is poorly expressed and has no apparent antiretroviral activity [21].

APOBEC3G, -F and -H, and mouse APOBEC3 are located in the cytoplasm of the cell, the location of poxviral replication. Most poxvirus genomes are relatively A/T-rich which could be a consequence of APOBEC3-induced mutational pressure [9]. It was, therefore, of interest to analyze whether VACV replication is affected by APOBEC3G. However, we could show that APOBEC3G, -F or -H, or mouse APOBEC3 expression has no effect on VACV replication. A limitation of our experiments might be the lack of a positive control, showing that APOBEC3 still confers an inhibitory function on retroviruses in the context of a VACV infection. However, the experiment is not doable. VACV infection leads to a shut down of cellular protein synthesis, which also inhibits retroviral particle formation. But we were able to show the inhibitory effect of APOBEC3G on retroviral and lentiviral vectors using the same experimental setup which did not result in an inhibition of VACV replication, confirming the significance of the study.

VACV has a very broad host range *in vitro *and is able to infect virtually all cell types [22]. Surprisingly, it has been shown recently that VACV tropism in hematopoietic cells is very restricted. Only poor infection of T lymphocytes, which express APOBEC3G, has been observed [23]. However, activated T-cells are permissive to VACV and our data support the concept that a receptor that permits VACV entry is missing from resting T cells [24].

Poxviruses have developed several strategies to evade the innate immune system [2]. Like HIV, which encodes Vif that induces degradation of APOBEC3G, VACV could encode a protein to overcome APOBEC3G. We investigated the protein levels of APOBEC3G during VACV infection and our results show that infection does not lead to a degradation of the protein. However, it cannot be excluded that VACV has evolved another mechanism to escape inhibition by APOBEC3G.

## Conclusion

Using transient transfections, we could show that APOBEC3G, -F or -H, or mouse APOBEC3 expression has no effect on VACV replication and VACV infection does not lead to a degradation of the APOBEC3G protein.

## Methods

### Plasmids and transfections

The following plasmids were used for transfections: pcDNA-APOBEC3G-MycHis encoding a C-terminally Myc-tagged human APOBEC3G [10], human APOBEC3F or -H [25], or mouse APOBEC3 [26] and the empty expression vector pRC-CMV. Retroviral vectors were generated by transfection of the plasmid pHIT60, encoding the MLV Gag/Pol region [11]; pEnv wt(HX), encoding the ecotropic MLV envelope protein [12] and pSFG-EGFP, a MLV-based retroviral vector encoding GFP [13]. Transfection of pHIT60, pEnv wt (HX) and pSFG-EGFP into 293T cell results in the production of infectious vector particles, which are able to transduce the GFP encoding vector sequences into target cells. Lentiviral vectors were generated by transient transfections using the following plasmids: pRRLsinCMV-GFPpre, pMDLg/pRRE, pRSVrev and a VSV-G envelope glycoprotein expression plasmid [27].

### Cell culture, transfections viral transduction and determination of titers

293T and NIH 3T3 cells were grown in Dulbecco's Modified Eagle's Medium (DMEM; Cambrex, Verviers, Belgium) supplemented with 10% fetal calf serum (FCS); (GIBCO/BRL, Eggenstein, Germany). HeLa, HeLa-APOBEC3G and BHK cells were grown in Roswell Park Memorial Institute-1640 Medium (RPMI-1640; Cambrex, Verviers, Belgium) supplemented with 10% FCS. RK-13 cells were grown in Eagle's Minimum Essential Medium (EMEM; Cambrex, Verviers, Belgium) supplemented with 10% FCS and 1% Non-Essential Amino Acids (Biochrom AG, Berlin, Germany).

To generate MLV-based vector particles, a day before transfection, cells were seeded at a density of 2 × 10^6 ^cells in a 10 cm tissue culture plate. The cells were transfected with 2 μg Gag/Pol expression plasmid (pHIT60) [11], 1 μg ecotropic MLV Env expression plasmid (pEnv [28]) and 3 μg GFP encoding vector plasmid [13] using the Fugene reagent (Roche, Penzberg, Germany). Either 2 μg APOBEC3G expression plasmid or the empty vector pRC-CMV was added in addition to assess the effect of APOBEC3G on vector titers. After two days of culture, serial dilutions of viral supernatants from transfected 293T cells were passed through 0.45-μm filters (Greiner, Frickenhausen, Germany) and incubated with 2 × 10^5 ^NIH 3T3 cells. 48–72 hours after transduction, the numbers of GFP-expressing cells were detected by FACS analysis. The titers are given in relative infectious units and are representative data of three independent experiments.

Lentiviral vectors were generated by seeding 293T cells at a density of 2 × 10^6 ^cells in a 10 cm tissue culture plate one day before transfection. The cells were transfected with the following expression plasmids [27] using the Fugene reagent (Roche, Penzberg): pRRLsinCMV-GFPpre, pMDLg/pRRE, pRSVrev and a VSV-G envelope glycoprotein expression plasmid. 48 hrs after transfection the vector supernatants were harvested, filtered and used for transductions. Titers were determined as described above for retroviral vectors.

For the analysis of VACV replication, cells (BHK or 293T) were transfected with 8 μg plasmid DNA, encoding APOBEC3 proteins and infected with vaccinia virus WR 48 h after the transfection. Cells were harvested at the indicated time points and vaccinia virus was titrated on RK-13 cells.

Transfection of APOBEC3G expression plasmid was analyzed by intracellular immunofluorescence staining with a mouse anti-Myc antibody (BD-Biosciences, Heidelberg, Germany) and a FITC-conjugated anti-mouse antibody after permeabilization of the cells with BD Perm/Wash Buffer (BD Pharmingen, Heidelberg, Germany). The numbers of FITC-stained cells were detected by FACS analysis.

### Western blot analysis

Cell lysates were obtained and Western blot analysis was performed as described previously [28]. Western blot analysis was performed with the following antibodies: mouse anti-6xHis antibody (Acris, Hiddenhausen, Germany), mouse anti-β-actin antibody (Sigma-Aldrich, Munich, Germany), polyclonal rabbit antiserum directed against the vaccinia virus E3 protein (kind gift of B. Jacobs) and horseradish peroxidase-coupled sheep anti-mouse IgG antibodies or protein A (Amersham Biosciences, Freiburg, Germany). Detection was performed using an enhanced chemiluminescence Western blot detection kit (Amersham Biosciences, Freiburg, Germany).

## Competing interests

The author(s) declare that they have no competing interests.

## Authors' contributions

MK, YS and YM-F performed the experiments. MK, YS, CM, GS and BS participated in the design of experiments, oversight of the conduction of the experiments, and in the interpretation of the results.
